# Adoptive Virus-Specific T Cell Therapy for Viral Infections After Allogeneic Hematopoietic Stem Cell Transplantation: Current Strategies and Future Perspectives

**DOI:** 10.3390/biom16101465

**Published:** 2026-10-08

**Authors:** Eva M. Weissinger, Lisa M. Schulz, Debora Basilio-Queiros, Michael Stadler, Penelope Kay-Fedorov

**Affiliations:** 1 Department of Haematology, Haemostasis, Oncology and Stem Cell Transplantation, Hannover Medical School, 30625 Hannover, Germany; schulz.lisamarie@mh-hannover.de (L.M.S.);; 2 German Center for Infection Research (DZIF, TTU-IICH), Hannover-Braunschweig Site, 30625 Hannover, Germany; kay-jackson.penelope@mh-hannover.de; 3 Miltenyi, 51429 Bergisch-Gladbach, Germany; 4 Institute of Virology, Hannover Medical School, 30625 Hannover, Germany

**Keywords:** immunocompromised host, hematopoietic stem cell transplantation, cytomegalovirus, viral infection, cellular therapies

## Abstract

Viral infections remain a major cause of morbidity and mortality after allogeneic hematopoietic stem cell transplantation (HSCT), particularly in patients with delayed immune reconstitution or prolonged immunosuppression. Although antiviral drugs have substantially reduced the incidence of viral disease, their use is limited by toxicity, resistance, and the inability to restore long-term antiviral immunity. Adoptive transfer of virus-specific T cells represents a targeted immunotherapeutic strategy to re-establish pathogen-specific immune responses while minimizing the risk of graft-versus-host disease (GvHD). This review focuses on the development and clinical application of adoptive virus-specific T cell therapy after allogeneic HSCT, with particular emphasis on cytomegalovirus (CMV). We summarize the evolution of adoptive antiviral T cell therapy, compare current manufacturing and selection strategies, and critically review available clinical evidence regarding efficacy, persistence, safety, and alloreactivity. In addition to CMV-directed products, we discuss approaches targeting Epstein–Barr virus (EBV), adenovirus (AdV), and other clinically relevant pathogens in the context of multipathogen-specific T cell products and third-party donor approaches. We further address current knowledge on immune monitoring, factors influencing antiviral immune reconstitution, as well as practical challenges that still limit broader clinical use. Finally, we discuss emerging strategies to improve product standardization, availability, and integration of adoptive T cell therapy with modern antiviral prophylaxis and graft-engineering approaches.

## 1. Introduction

### 1.1. Allogeneic HSCT: Advances and Remaining Challenges in Immune Reconstitution

Allogeneic hematopoietic stem cell transplantation (HSCT) is the only curative treatment for hematologic malignancies in adults. Over the seven decades since the first successful HSCT, major progress has been achieved [1,2,3]. Improved human leukocyte antigen (HLA)-typing, donor selection, and prophylactic and preemptive therapy of infectious complications made HSCT more effective [4]. Reduction of the toxicity of conditioning regimens, improved graft-versus-host disease (GvHD) prophylaxis, and the transfer of donor leukocytes (DLI) as treatment or prophylaxis of relapse allowed broader application of HSCT. These improvements led to higher survival rates after HSCT, even in patients with aggressive diseases such as acute leukemia (AL). Reduced toxicity allowed transplantation of elderly patients and patients with higher comorbidity scores. However, serious complications do still limit the application of HSCT. The most serious complication with the largest single cause of mortality is recurrence of the primary disease. About 50% of mortality is due to leukemia relapse [5,6,7]. Acute and chronic GvHD are the leading causes of non-relapse mortality (NRM) and contribute to about 25% of transplant-related mortality (TRM) [8,9]. GvHD results from an allogeneic reaction of donor T cells against healthy tissues of the recipient, triggered by major and/or minor histocompatibility antigen (mHag) differences between recipient and donor [10]. Opportunistic infections occur during periods of immunosuppression and become particularly relevant when immunosuppression is intensified, for example, during the treatment of severe acute or chronic GvHD. Latent viruses, including cytomegalovirus (CMV), Epstein–Barr virus (EBV), human herpes virus 6 (HHV-6), and others, are the major contributors to morbidity and, in some cases, mortality, followed by fungal infections and bacterial sepsis. One of the biggest challenges of HSCT is to achieve a balance between tolerance and rejection, as well as between relapse prevention and graft-versus-leukemia (GvL) effects on the one hand and severe GvHD and serious infections on the other.

Donor leukocytes initially served as a treatment for relapse of chronic myeloid leukemia (CML), but their beneficial effects in controlling viral infections and avoiding graft rejection became clear [11,12,13]. DLI is a mixture of leukocytes containing T, B, and NK cells as well as monocytes and limited numbers of dendritic cells. A major complication of DLI is GvHD, with the associated need for immunosuppressive treatment, which in turn increases the risk of viral reactivation and, additionally, disease relapse. The introduction of DLI as a post-transplant treatment of HSCT made clear that immune modulation after HSCT is essential for increased relapse–free and overall survival of transplant recipients. Peripheral blood stem cell transplantation (PBSC) allowed for CD34^+^ enrichment in the late 1990s [14]. In HSCT, CD34^+^ enrichment, either in vivo or ex vivo T cell depletion, had the potential to reduce the incidence and severity of acute GvHD [15]. However, while CD34-enrichment was successful in reducing GvHD, it also led to increased rates of graft rejection, viral reactivation, and disease relapse [16]. The beneficial effects of CD34^+^ enrichment appear to be particularly pronounced in haploidentical HSCT, where improved overall survival of the patients has been reported. In the late 1990s, T cell repletion using genetically modified donor T cells was investigated in European clinical trials. These approaches mainly used herpes simplex thymidine kinase (HSV-Tk) as a safety switch, allowing T cell depletion in case of GvHD development, with the aim of reducing the risk of severe acute GvHD [17,18,19]. Others used T cell repletion strategies without gene transfer, starting with small doses of T cells early after HSCT and slowly increasing doses in the absence of GvHD [20]. DLI therefore profits from both the GvL and antiviral effects of donor T cells [21,22]. Specific T cell clones and T cell lines have been developed and successfully used for the treatment of both disease relapse and post-transplant infections [23,24,25]. To understand why virus-specific T cell therapy has become an important therapeutic approach after HSCT, it is first necessary to consider the infectious complications that occur during immune reconstitution.

### 1.2. Infectious Complications After Allogeneic HSCT

Opportunistic infections are serious complications that affect the morbidity and mortality of transplant patients [26]. Important risk factors include immunodeficiency and mucosal injury caused by the pre-transplant conditioning regimen [27], T cell-depleted allogeneic transplantation [28], delayed immune reconstitution due to HLA differences between donor and host as well as immunosuppressive therapy for GvHD [29]. Prophylaxis and treatment of transplant patients with medication is effective, either by killing the pathogens or controlling their replication. However, viral infections or invasive fungal infections may be refractory to treatment due to drug resistance or treatment-limiting toxicities [30,31]. The most common infections in immunocompromised patients are caused by viral, bacterial, parasitic, and fungal pathogens. Most of these pathogens are effectively controlled by the immune system in healthy individuals, in whom infections are often asymptomatic or cause only mild disease. In HSCT patients, however, infection or reactivation can lead to rehospitalization, prolonged morbidity, and even to reduced overall survival [32,33]. Antibiotics, antifungals, and antiparasitic and antiviral medications have substantially improved the prevention and treatment of infectious complications after HSCT. Nevertheless, these approaches have important limitations, including drug-related toxicities. Although prophylactic and preemptive therapies, such as letermovir and ganciclovir, respectively, effectively reduce the risk of viral disease, durable protection against recurrent reactivation and invasive infections ultimately depends on the reconstitution of pathogen-specific T cell, B cell, and NK cell immunity [34,35,36]. In addition, prophylactic treatment, for example with letermovir, can hinder the development of pathogen-specific immune responses, allowing infection or reactivation to occur, albeit at a later stage of recovery after HSCT [37].

CMV, EBV, and adenovirus (AdV) are among the major viral pathogens leading to complications post-HSCT. Other critical non-viral infections include invasive fungal infections, which are mainly caused by pathogens such as *Aspergillus* or *Candida*. Invasive mold infections remain one of the leading causes of mortality in allo-HSCT [38].

### 1.3. CMV Reactivation and Monitoring of Antiviral Immune Reconstitution

In the following section, we will focus on CMV, a herpes virus that typically produces mild or no symptoms upon initial infection in immunocompetent individuals and subsequently persists lifelong in the host, with sporadic reactivations that often remain unnoticed [39,40]. In immunocompromised people, however, CMV infection and reactivation can have serious consequences. CMV seroprevalence varies significantly according to geographic, socioeconomic, and demographic factors and generally increases with age, with higher rates observed in lower-income settings [41]. CMV persists lifelong in infected individuals in certain blood cells as well as endothelial and epithelial cells. Reactivations are controlled primarily by T cells specific for CMV [42].

The CMV serostatus of both donor and recipient has a profound effect on the risk and clinical outcome of CMV reactivation after HSCT. For example, CMV-seropositive (CMV+) patients receiving a graft from a CMV-seronegative (CMV-) donor have the highest risk of clinically significant CMV reactivation with prolonged complications [43,44]. The most common manifestations of CMV disease include gastrointestinal disease, pneumonia/interstitial pneumonitis, hepatitis, retinitis, and encephalitis. Additionally, several studies have reported a correlation between CMV reactivation and the onset or aggravation of GvHD [45,46,47]. Treatment of HSCT patients with CMV reactivation and GvHD is challenging, since the immunosuppressive therapy required to control GvHD further increases the risk of CMV reactivation. Conversely, reducing immunosuppression to allow better CMV control may increase the risk of recurrent GvHD. CMV seropositivity can, however, have beneficial effects on post-transplant outcomes. We and others have reported that CMV-seropositivity may be associated with a reduced risk of relapse, especially in the presence of at least one CMV-specific cytotoxic T lymphocyte (CMV-CTL)/µL in peripheral blood [48,49].

Monitoring immune reconstitution using either tetramer staining or a cytokine capture assay for virus-specific CTLs is essential for identifying patients with quantitative or qualitative deficiencies in CMV-specific T cell immunity who may benefit from adoptive T cell transfer. In addition, this type of monitoring allows clinical follow-up data to be linked with CMV reactivation and the presence of CMV-CTLs. These observations have provided the rationale for therapeutic strategies aimed at restoring virus-specific immunity through the adoptive transfer of donor-derived T cells.

### 1.4. Development of Adoptive Antiviral T Cell Therapy

In 1992 and 1995, Greenberg and colleagues published the first studies investigating the prophylactic transfusion of in vitro expanded CMV-specific CD3^+^/CD8+ T cell clones for protection against CMV reactivation [23,50]. Following these initial studies, many researchers developed approaches to enrich T cell subpopulations reactive to virus lysate or to establish virus-specific T cell lines/clones. Einsele and colleagues were one of the first groups to report the safe and efficient transfer of CMV-specific T cells expanded in the presence of dendritic cells (DCs) loaded with virus-infected cell lysate or peptides [24].

Since 2000, the safety of transplantation protocols has increased significantly by reducing the toxicity of the conditioning regimen (reduced intensity conditioning, RIC). Around this time, it became possible to isolate antigen-specific T cells directly ex vivo based on antigen-induced secretion of interferon-γ (IFN-γ) using cytokine-capture assays followed by selection with magnetic microbeads, thereby eliminating the need for prolonged ex vivo expansion. This approach enabled the selection of both CD3^+^/CD4+ and CD3^+^/CD8+ T cells. Over the last few decades, several cellular treatment options have been developed and successfully implemented. Nevertheless, cellular therapy is still associated with several challenges; complex manufacturing processes and the resulting high costs, in particular, limit the broad implementation of cellular transfer into clinical routine. In the following sections, we focus primarily on CMV as the best-characterized model for adoptive antiviral T cell therapy after allogeneic HSCT. Evidence from EBV, AdV, and other pathogen-specific or multipathogen-specific T cell approaches is discussed where it provides complementary insights into manufacturing, safety, clinical efficacy, and broader implementation.

Over the last 25 years, adoptive T cell therapy has emerged as a potential alternative to pharmacologic treatment for patients with refractory post-transplant infections [51,52,53]. DLI is a treatment option largely used to prevent or treat relapse in HSCT patients, but severe GvHD unfortunately remains a concomitant risk [54,55]. In the early 1990s, it became evident that DLI was also beneficial for the treatment of viral infections due to the presence of virus-specific memory T cells among lymphocytes from seropositive donors [12]. Despite these considerable benefits, the treatment of viral infections with DLI had limitations in both safety and efficacy, mainly due to the high frequency of allo-reactive T cells and the low frequency of antigen-specific T cells [23,56]. These limitations led to efforts to enrich antigen-specific T cells and/or to deplete allo-reactive T cells, with the aim of enhancing pathogen-specific immune reconstitution while reducing the risk of GvHD. One of the pioneering studies by Ridell et al. demonstrated successful reconstitution of virus-specific T cell immunity in HSCT patients at high risk of developing CMV disease following the prophylactic transfusion of in vitro expanded CMV-specific CD8+ T cell clones [23]. Although this approach successfully restored CMV-specific immunity, the in vitro expansion of virus-specific T cell clones had several drawbacks for integration into routine clinical practice. Since then, innovative technological developments and novel basic immunological findings have led to improvements and advances in the uptake of the treatment of infectious diseases using adoptive anti-pathogen T cell transfer.

## 2. Generation of Antiviral T Cell Products

### 2.1. In Vitro GMP Manufacturing of Antiviral T Cell Products

There are two main strategies for the generation of good manufacturing practice (GMP)-grade antiviral T cell products: the expansion of virus-specific T cells and the direct selection of antigen-specific T cells from donor blood. The first approach relies on repetitive in vitro stimulation of donor blood cells with viral antigens and antigen-presenting cells to produce virus-specific T cell clones or lines [23,57]. A main disadvantage of this type of cellular product is the long and laborious preparation process of several weeks. An alternative approach became possible with the development of magnetic selection methods that allow rare virus-specific T cells to be isolated directly from peripheral blood. These methods are based either on IFN-γ secretion (cytokine capture assay, Feuchtinger et al.) or peptide/MHC multimer labeling, allowing a significant reduction in the preparation time of the cellular product under GMP conditions to just one to two days [58].

**Figure 1:**
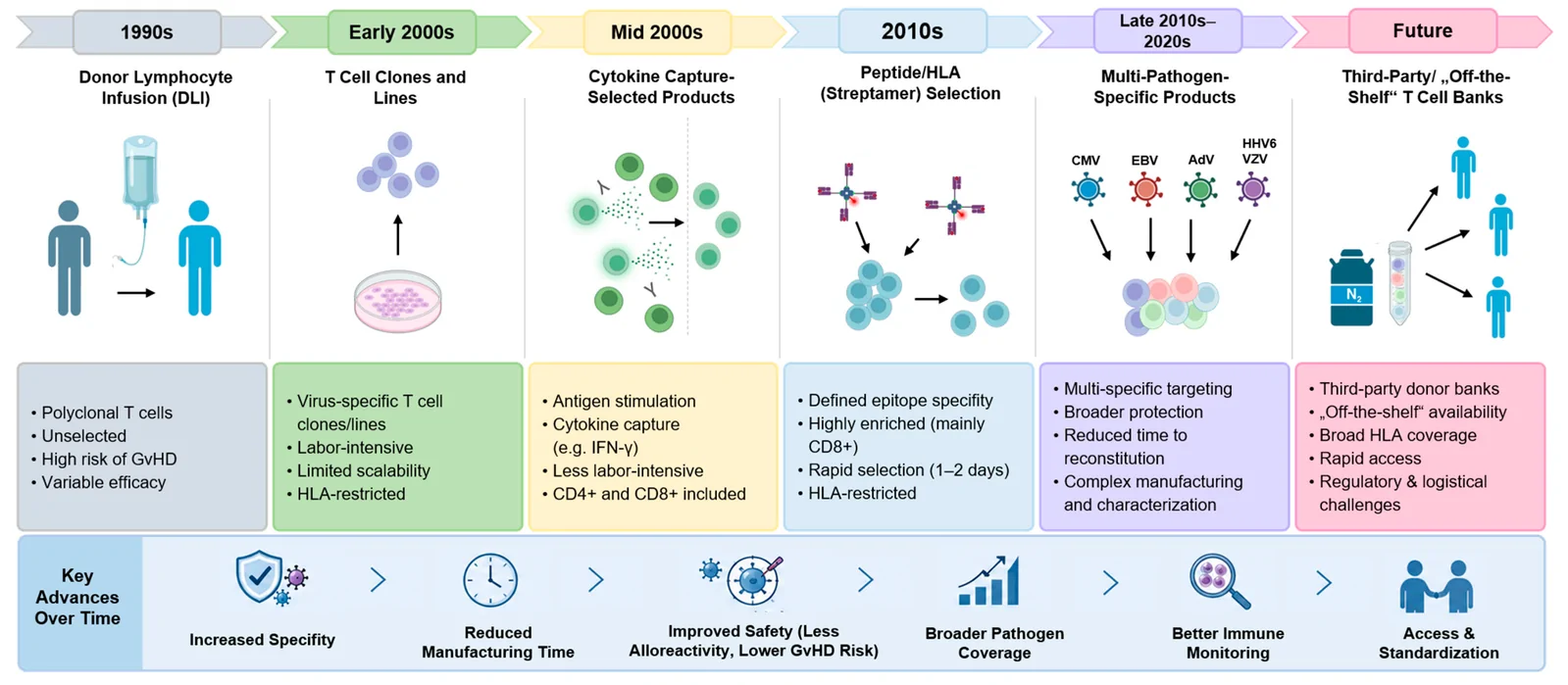
Evolution of adoptive antiviral T cell therapy. Schematic overview of the major developments in adoptive antiviral T cell therapy, from donor lymphocyte infusion (DLI) and virus-specific T cell clones or lines to antigen-specific selection approaches, including cytokine capture and peptide/MHC multimer- or Streptamer-based selection. More recent strategies include multipathogen-specific T cell products and third-party donor-derived T cell banks, aiming to reduce manufacturing time and enable broader and more readily available treatment options. The figure illustrates the evolution from individually generated, extensively expanded products toward rapidly selected, multipathogen-specific, and potentially off-the-shelf cellular therapies. Abbreviations: AdV, adenovirus; BKV, BK virus; CMV, cytomegalovirus; DLI, donor lymphocyte infusion; EBV, Epstein–Barr virus; GvHD, graft-versus-host disease; HLA, human leukocyte antigen; IFN, interferon; VZV, varicella zoster virus.

The cytokine capture assay requires stimulation of peripheral blood samples with viral antigens, such as peptides or proteins, for about 4 to 16 h to induce IFN-γ production by virus-specific T cells. Antigen-activated T cells are incubated with a capture matrix that immobilizes secreted IFN-γ on the cell surface. Subsequently, the IFN-γ-producing T cells are selected via microbeads coated with anti-IFN-γ antibodies. Peptide/MHC multimer technology, in contrast, allows the selection of peptide-specific CD8+ T cells via binding of peptide-MHC multimers to cognate T cell receptors (TCR), followed by magnetic enrichment. This method does not require any prior antigen stimulation or ex vivo expansion of peripheral blood cells before selection. Both cytokine capture and peptide/MHC selection enable rapid and effective production of antigen-specific T cells in a very short time, and both are equally compatible with GMP production. However, the two approaches differ in their applicability. Whereas the number of available peptide/MHC multimer reagents is limited to the most common HLA/epitope pairs, the cytokine capture assay allows isolation of virus-reactive T cells independent of HLA allotypes. A disadvantage of the cytokine capture assay compared to peptide/MHC multimer technology is the need for a short ex vivo antigen-stimulation step. At the same time, this step offers greater flexibility, as the antigen used for stimulation can be selected according to the patient’s clinical needs. A broad range of stimulating antigens can be used, including individual peptides, peptide pools, proteins, or combinations of viral antigens. A broad panel of viral protein antigens is now available as premixed GMP-grade peptide cocktails, covering a wide range of CD4+ as well as CD8+ T cell epitopes across multiple HLA alleles. Although these rapid manufacturing technologies have considerably improved the availability of virus-specific T cell products, their clinical application depends not only on manufacturing efficiency but also on their safety profile. The principal manufacturing and selection platforms currently used for the generation of virus-specific T cell products differ substantially with regard to starting material, production time, HLA dependence, product composition, and clinical applicability (Table 1). Overall, the development of these technologies reflects a transition from labor-intensive expansion of virus-specific T cell lines toward more rapid antigen-specific selection and, more recently, the generation of multipathogen-specific and third-party T cell products (Figure 1).

### 2.2. Alloreactivity: Product Composition and Safety

#### 2.2.1. Product Composition: CD4+ Versus CD8+ T Cells

Over the past three decades, techniques for the isolation and generation of pathogen-specific T cells under GMP conditions have significantly improved. These approaches allow the isolation of both pathogen-specific CD4+ and CD8+ T cells using the cytokine capture system [59] or the Streptamer-based selection of CD8+ T cells restricted by defined HLA alleles and virus-specific peptides [60]. Despite these advances, several issues still need clarification before this approach can become standard clinical practice. In particular, contradictory data on the protective role of CD4+ and CD8+ T cells left unresolved whether adoptive T cell products should contain both subsets or primarily CD8+ T cells. Several studies support a critical role for CD4+ T cells in antiviral immunity. Prophylactic infusion of CMV-specific CD4+ T cells in patients lacking CMV-specific T-helper responses increased CMV-CTL frequencies in both CD4+ and CD8+ T cell subpopulations and resulted in successful viral clearance [24]. Subsequent studies by Riddell et al. and Walter et al. demonstrated that transfused CMV-specific CD8+ T cell clones progressively declined in patients lacking CD4+ T cells [23,50]. Consistent with these findings, subsequent studies have demonstrated the critical role of CD4+ T cells in both maintaining the functionality of cytotoxic CD8+ T cells and directly fighting the viral infection [61,62]. On the other hand, the transfusion of CMV-specific CD8+ T cells alone has also been proven efficient in clearing the viremia and increasing the frequency of donor-derived CMV-specific CD8+ T cells as well as in recruiting CD4+ T cells in recipients [63]. In a multicenter study, Leen et al. observed that transfusions of either CD4+ or CD8+ T cells were equally protective against viral infections [64]. The inclusion of CD4+ T cells remains the most controversial issue for adoptive immunotherapy, however, as several studies have reported a higher alloreactive potential of this T cell subset [65,66].

#### 2.2.2. In Vitro Alloreactivity Versus Clinical GvHD Risk

Although the allogeneic reactivity of pathogen-specific T cells has been thoroughly investigated, their potential to induce clinically relevant GvHD still needs to be clarified, particularly when HLA-mismatched donors are used. Several in vitro studies have reported the cross-reactive potential of expanded virus-specific T cells toward allogeneic HLA antigens [67,68,69,70,71]. Single-virus-specific CD4+ and CD8+ T cell lines or clones directed against CMV, EBV, VZV, and influenza virus have been shown in vitro to recognize and lyse cells expressing allo-HLA class I and class II molecules, including normal cell subsets [67]. Long-term culture and repeated antigenic stimulation during the generation of T cell clones may contribute to the alloreactivity observed in these experimental settings.

In the clinical setting, however, this alloreactivity has not been reported, including in selected HLA-mismatched clinical settings [68]. This discrepancy may be due to differences between long-term expanded T cell clones used in experimental studies and antigen-selected products used in clinical protocols. The latter are typically generated with shorter ex vivo manipulation and contain an enriched population of virus-specific T cells. In products generated using the cytokine capture system (CCS), alloreactivity could be decreased by 1.27 log (mean value after three independent experiments) following the selection and expansion of AdV-specific T cells using the CliniMACS CCS IFN-γ system [72]. Comparable results were shown for CMV-specific T cells, with a 95% reduction in alloreactivity following antigen-specific selection [73].

One limitation of the data above is that alloreactivity was generally not assessed against recipient-derived target cells. In clinical practice, such testing is not feasible because of the limited time available before treatment and the practical challenges of obtaining representative target cells from organs commonly affected by GvHD, including the skin, gastrointestinal tract, and liver.

A distinction should therefore be made between in vitro evidence of alloreactivity and clinically observed GvHD after adoptive transfer of virus-specific T cells. Detection of alloreactivity in vitro does not necessarily predict clinically relevant tissue damage or the development of GvHD after infusion. Antigen-specific enrichment reduces the proportion of nonspecific donor T cells, and the transferred cells may require appropriate antigenic stimulation for substantial in vivo expansion. In addition, in vitro target-cell assays cannot fully reproduce the complex conditions governing tissue homing, local inflammation, and immune activation in vivo. Nevertheless, the currently favorable clinical safety profile should not be interpreted as proof that alloreactivity is irrelevant. Rather, the potential risk of clinically relevant alloreactivity should be considered in relation to the individual product and clinical setting, particularly in HLA-mismatched and third-party settings.

In a recent study from our group, CMV-CTLs isolated by IFN-γ secretion assay and subsequently expanded in vitro did not induce relevant cutaneous tissue damage in an in vitro skin explant model while maintaining high antiviral activity [74]. At low cell doses (5 × 10^5^), none of the CMV-CTLs led to GvH reactions in the HLA-mismatched recipient’s skin, whereas at the high cell dose (1 × 10^6^), two of nine CMV-CTL preparations induced mild GvH-associated skin damage. These reactions were associated with higher CD4+ T cell frequencies in the respective lines. These findings provide additional experimental evidence that the alloreactive potential of virus-specific T cell products may depend on both product composition and cell dose. At the same time, they underline the limitations of extrapolating from experimental models to clinical GvHD risk and support the need for product characterization and clinical monitoring.

### 2.3. Risk Mitigation and Safety Monitoring

Taken together, the available experimental and clinical data support a favorable clinical safety profile of antigen-selected virus-specific T cell products. Nevertheless, risk assessment remains particularly important in HLA-mismatched and third-party settings. Relevant factors include donor selection and HLA compatibility, product composition and antigen specificity, the intended cell dose, and, where feasible, functional testing for alloreactivity. Following infusion, patients should be monitored for GvHD as well as viral load and virus-specific immune reconstitution. Key elements of risk mitigation are summarized in Table 2.

It is also important to correlate the phenotype and functionality of the infused cells with the clinical outcome. The following section therefore discusses clinical responses, including viral clearance and the kinetics of virus-specific immune recovery, following cellular therapies based on the cytokine secretion and Streptamer selection technologies.

## 3. Clinical Application of Virus-Specific T Cell Therapy

**Figure 2:**
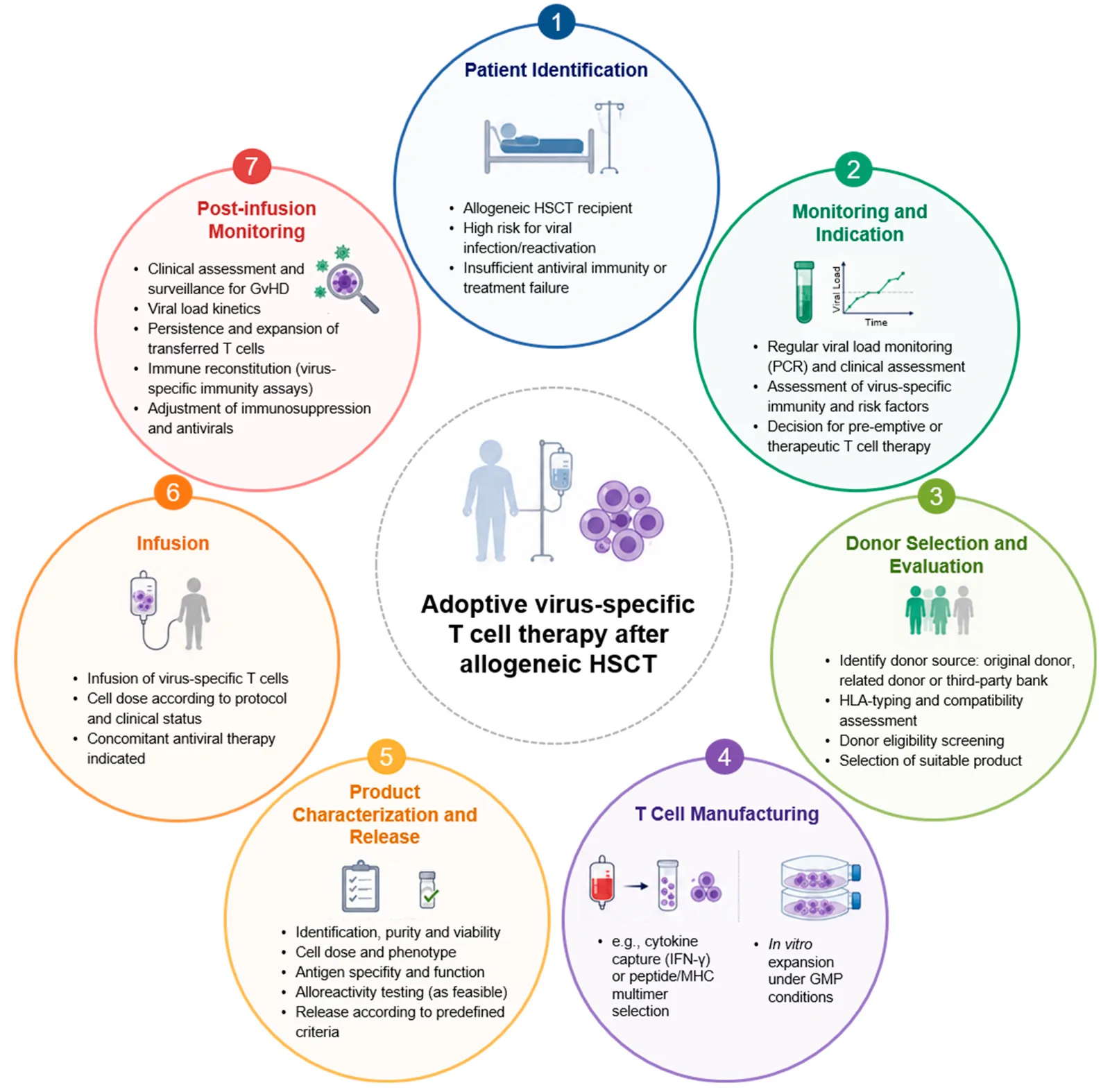
Clinical workflow for adoptive virus-specific T cell therapy after allogeneic HSCT. Schematic representation of the potential clinical workflow from patient identification and viral and immune monitoring to donor or product selection, GMP-compliant T cell manufacturing, product characterization and release, infusion, and post-infusion monitoring. Depending on the clinical setting, therapy may involve donor-derived or third-party virus-specific T cell products and may be administered alongside antiviral therapy. Post-infusion assessment includes monitoring of viral load, GvHD, persistence of transferred T cells, and virus-specific immune reconstitution. Abbreviations: GMP: good manufacturing practice; GvHD: graft-versus-host disease; HLA: human leukocyte antigen; HSCT: hematopoietic stem cell transplantation; IFN: interferon.

The clinical implementation of adoptive virus-specific T cell therapy involves several consecutive steps, including viral and immune monitoring, patient selection, donor or product selection, manufacturing and product release, infusion, and subsequent assessment of viral and immunological responses (Figure 2).

### 3.1. Cytokine Capture Assay-Based Selection–Clinical Trials

Several studies have made use of the IFN-γ capture assay to select antigen-specific T cells. Feuchtinger and colleagues published the clinical data from 13 patients treated at multiple centers across Germany with an infusion of pp65-specific IFN-γ-secreting CD4+ and CD8+ T cells for refractory CMV infection or CMV disease after HSCT [75]. The cellular products contained naïve, central memory, and effector memory cells. In vivo expansion of the transferred cells correlated with clearance or significant reduction in viremia and was observed for both the CD4+ and CD8+ subsets. Transferred T cells could be detected in vivo for an average of 3–6 weeks after infusion, and, in some patients, pp65-specific immunity persisted for more than 6 months [75].

Moosmann and colleagues used the IFN-γ capture assay following stimulation with EBV-derived peptides to generate EBV-specific T cells to treat post-transplant lymphoproliferative disease induced by EBV [76]. Three of six patients achieved complete and stable remission following combined treatment with rituximab, an anti-CD20 antibody, and low numbers of CD4+ and CD8+ EBV-specific T cells. Non-responders suffered from late-stage disease with multiorgan dysfunction at the time of T cell transfer. In two responders, transferred EBV-specific T cells underwent rapid in vivo expansion, remained detectable at high levels for approximately six months, and subsequently contracted to stable levels comparable to those observed in healthy individuals. This expansion and contraction pattern is characteristic of antigen-specific T cell responses and was associated with protection for at least two years after transfer. Detailed analyses early after transfer revealed that EBV-specific T cells had an effector memory phenotype, which after contraction evolved toward central memory or terminally differentiated effector cells [76]. The use of a combination of immunodominant antigens from different viruses also allowed simultaneous selection of CMV-, EBV-, and AdV-specific CD4+ and CD8+ T cells, extending the applicability of the cytokine-capture approach to multivirus-specific T cell products [77].

### 3.2. Peptide/MHC Multimer (Streptamer)-Based Selection–Clinical Trials

The development of “reversible” TCR staining using Streptamers allowed the selection of phenotypically and functionally intact cells [58]. This technique has subsequently been used for adoptive T cell transfer, since it has been shown that antigen-specific CD8+ T cells selected with Streptamers induce long-lasting immune responses without increasing the risk for GvHD. Schmitt and colleagues reported the treatment of two patients for recurrent CMV antigenemia after HSCT using CMVpp65-specific, Streptamer-selected T cells [63]. The infused cells had an effector/memory phenotype and were capable of secreting IFN-γ upon in vitro stimulation. They rapidly expanded in vivo, acquired an effector phenotype, and cleared CMV reactivation without any signs of GvHD [63]. Odendahl and colleagues further demonstrated the potential of Streptamer-selected T cells to restore antiviral immunity in patients with refractory CMV viraemia. In this study, 22 cell products displayed excellent viability, cytotoxicity and purity, with effectively removed selection reagents [60]. More recently, GMP-compliant Streptamer technology was implemented to enrich EBV- and AdV-specific T cells. Because EBV- and AdV-specific T cells occur at very low frequencies in peripheral blood, the purity of large-scale products was limited, reaching 44% and 6.7%, respectively, among CD3+ cells [78]. Higher purity could, however, successfully be achieved by small-scale selection or simultaneous application of EBV- and AdV-Streptamers. Most products displayed antigen-specific IFN-γ responses and were enriched for effector-memory and central-memory phenotypes, supporting their suitability for clinical application [78].

Overall, clinical trials to date have confirmed the safety and efficacy of the adoptive transfer of virus-specific T cells. A comparative overview of representative clinical studies is provided in Table 3. However, controlled clinical trials, involving the tracking of the infused cells, are required in order to further investigate the optimal conditions for successful and long-lasting effects of the adoptive transfer. Despite encouraging clinical experience, several challenges remain before adoptive virus-specific T cell therapy can be broadly implemented in routine clinical practice.

## 4. Future Perspectives

### 4.1. Next-Generation T Cell Products

Adoptive transfer of virus-specific T cells has emerged as a promising strategy for restoring antiviral immunity in immunocompromised patients, particularly after allogeneic HSCT. An important future direction is the development of multipathogen-specific T cell products targeting several clinically relevant viruses simultaneously. Such products may be particularly useful after HSCT, where patients are susceptible to reactivation or infection with multiple pathogens, including CMV, EBV, and AdV. Several strategies have been developed to simultaneously generate T cells specific for multiple viral and, more recently, fungal pathogens. Initial studies focused on CMV, EBV, and AdV and used the clinical-grade adenovirus vector Ad5f35 encoding viral antigens such as CMV pp65 to transduce antigen-presenting cells (APCs), including dendritic cells or EBV-transformed B cells. This enabled efficient stimulation and expansion of virus-specific T cells [67,79,80]. Although effective, these approaches required the genetic modification of APCs.

To overcome this limitation, a new enrichment strategy based on activation-induced transient CD154 (CD40L) expression has been exploited to isolate antigen-reactive T cells, particularly CD4+ T cells and, to a lesser extent, CD8+ T cells. This approach enables the production of multipathogen-specific T cell preparations without genetic modification of APCs. Alloantigen-depleted CD4+ and CD8+ T cell lines could be generated within 14 days, with high specificity for the most common post-transplantation pathogens, together with substantial proliferative capacity and preserved in vitro functionality [81].

More recently, DNA plasmids or peptide pools have been used to pulse APCs, thereby avoiding safety and regulatory issues associated with viral-vector transduction. Combining the “pepmix” (peptide mixture) approach or plasmid-transfected DCs with expansion in gas permeable rapid-expansion (G-Rex) bioreactors provided further advances, increasing both feasibility and applicability of T cell therapy. These rapidly (10–12 days) expanded multivirus-specific T cells provided effective antiviral protection in clinical trials [79,82].

Beyond increasing the number of targeted pathogens, future development will need to focus on the composition and functional quality of these cellular products. Multipathogen-specific products may reduce the need to manufacture separate products for individual infections, but broader pathogen coverage also increases manufacturing and characterization requirements. The frequency, specificity, and functional activity of individual pathogen-specific T cell populations therefore need to be carefully assessed.

A further challenge is the definition of appropriate potency assays and release criteria. Current virus-specific T cell products differ in donor source, antigen stimulation, manufacturing platform, cellular composition, and antigen specificity. Standardized criteria for identity, purity, specificity, viability, and functional potency would facilitate comparison between clinical studies and support broader clinical implementation. Because no single assay is likely to fully capture the complex in vivo activity of a cellular product, a combination of phenotypic and functional measures, including antigen-specific activation, cytokine production, cytotoxicity, or antiviral activity, may be required.

### 4.2. Broadening the Use of Immunotherapy

Several barriers prevent the broader use of virus-specific T cell therapies after HSCT, including the use of pathogen-naïve donors and umbilical cord blood grafts. To address this limitation, several strategies have been developed. One approach involves the in vitro priming of donor-derived cells using antigen-pulsed or genetically modified APCs [83]. Another option is the transfer of virus-specific TCR genes into donor primary T cells using viral vectors [84]. Antigen-specific responses in recipients can also be boosted by vaccination with peptide-loaded donor-derived DCs [85].

Apart from the above-mentioned strategies, the selection of virus-specific T cells from healthy, seropositive third-party donors is another attractive alternative. Haque and colleagues showed for the first time that partially matched third-party EBV-CTLs led to the control of post-transplant lymphoproliferative disorder after solid organ transplantation [86]. Subsequent studies confirmed successful post-HSCT treatment of refractory CMV, EBV, and AdV infections using third-party virus-specific T cells [83,87]. These findings initiated the idea of donor registries and biobanks with cryopreserved antigen-specific T cells, which could provide an “off-the-shelf” immunotherapy product.

Third-party and banked T cell products could substantially reduce the time required for patient- or donor-specific manufacturing and may therefore be particularly valuable in patients with rapidly progressive viral disease. Their broader use, however, remains dependent on HLA compatibility and the HLA restriction of the virus-specific T cell population. Establishing broadly applicable donor banks will consequently require sufficient HLA diversity and careful donor selection to achieve adequate population coverage. In addition, although third-party T cells can mediate effective antiviral responses, the extent and duration of their in vivo expansion and persistence, as well as their contribution to long-term immune reconstitution, remain incompletely understood.

In parallel, rapid manufacturing and magnetic enrichment technologies have also expanded the application of adoptive T cell therapy in HSCT patients with refractory CMV, EBV, and AdV infections [75,76]. More recently, adoptive T cell therapy has been extended to other viral infections, including varicella zoster virus, BK virus, or human herpesvirus 6 [88,89], as well as invasive fungal infections caused by *Aspergillus* or *Candida* [90]. This broader pathogen coverage expands the potential clinical applications of pathogen-specific cellular therapy beyond the most extensively studied viruses but also increases the challenge of developing standardized products for less common or rapidly evolving pathogens.

Regulatory, logistical, and economic factors will also influence broader implementation. GMP-compliant manufacturing, donor screening, quality control, cryopreservation, storage, and distribution require specialized infrastructure. Centralized or regional cell banks and standardized manufacturing procedures may facilitate access beyond specialized transplantation centers. Prospective health-economic analyses are needed to determine in which clinical settings adoptive T cell therapy provides a cost-effective alternative or complement to conventional antiviral treatment.

Long-term efficacy remains another challenge. Persistence and functionality of transferred T cells likely depend on their differentiation state, antigen exposure, immunosuppression, donor-recipient compatibility, and the recipient’s endogenous immune recovery. Future clinical studies should therefore assess not only short-term viral clearance but also the durability of antiviral responses and long-term persistence of transferred cells.

Another potential limitation is viral immune escape. Products targeting a restricted number of viral epitopes may be affected by viral sequence variation or loss of target antigens. Targeting multiple antigens and maintaining broader T cell repertoires may reduce this risk, although this may increase manufacturing complexity. A better understanding of pathogen diversity, antigen selection, and mechanisms of immune escape will therefore be important for the design of future products.

The role of adoptive T cell therapy also needs to be considered in the context of contemporary antiviral prophylaxis and treatment. In particular, the introduction of letermovir has substantially changed the prevention of CMV infection in high-risk patients after allogeneic HSCT. While prophylaxis reduces the incidence of clinically significant CMV reactivation, its effects on CMV-specific immune recovery and the best timing of immune-based therapies are still unclear. Virus-specific T cell therapy may complement antiviral treatment in patients with recurrent or refractory infections, antiviral resistance, drug intolerance, or delayed immune reconstitution. Combining T cell therapy with antiviral prophylaxis and viral and immune monitoring may improve the management of viral infections after allogeneic HSCT.

## 5. Conclusions

Adoptive transfer of virus-specific T cells has evolved from an experimental concept into a promising immunotherapeutic strategy for the treatment of viral infections after allogeneic HSCT. By restoring pathogen-specific immunity rather than solely suppressing viral replication, this approach may provide more durable antiviral protection than conventional antiviral treatment alone. Despite encouraging clinical results, several challenges remain. Further development will require a better understanding of the mechanisms of action of virus-specific T cells. Improved biomarkers and immune monitoring strategies are also needed to better predict therapeutic responses, persistence of transferred T cells, and immune competence following allogeneic HSCT. At the same time, standardized product characterization, potency testing, and release criteria are needed to ensure consistent manufacturing and facilitate comparison between clinical studies. Regulatory requirements and the costs and logistics of GMP-compliant manufacturing and distribution remain important barriers to clinical implementation. The combination of antiviral prophylaxis, such as letermovir for CMV, viral load monitoring, immune monitoring, and appropriately timed virus-specific T cell therapy may allow a more individualized approach to infection prevention and treatment. Further improvements in graft engineering, including strategies such as CD34-positive selection, TCRαβ depletion, and CD45RA depletion, may further reduce transplant-related complications while preserving antiviral immunity. Together with standardized, readily available, and pathogen-specific T cell products, these approaches are expected to further improve immune reconstitution and clinical outcome after allogeneic HSCT.

## Figures and Tables

**Table 1:** Comparison of major manufacturing and selection strategies for virus-specific T cell products.

Strategy	Starting Material	Antigen Stimulation	HLA Restriction	Predominant T Cell Composition	Typical Manufacturing Time	GMP Feasibility	Main Advantages	Main Limitations	Clinical Applicability
In vitro expansion of virus-specific T cell lines/clones	Donor PBMCs or T cells	Repeated stimulation with viral antigens, peptides, APCs, or virus-transformed cells	Depends on antigen-presentation strategy	CD4+ and/or CD8+	Several weeks	Yes, but complex	High cell numbers; broad antigen specificity; multipathogen products possible	Labor-intensive; long manufacturing time; risk of phenotypic changes during culture; limited suitability for urgent treatment	Established proof-of-concept and clinical use; suitable for planned or banked products
IFN-γ cytokine capture assay (CCS)	Donor PBMCs	Short antigen stimulation with peptides, peptide pools, proteins, or antigen preparations	Not restricted to predefined HLA/epitope pairs	CD4+ and CD8+	Approximately 1–2 days	GMP-compatible	Rapid generation; broad antigen flexibility; enrichment of functional CD4+ and CD8+ cells	Requires sufficient precursor frequency and short ex vivo stimulation; product composition may vary	Clinical application for CMV, EBV, AdV, and multipathogen products
Peptide/MHC multimer or Streptamer selection	Donor PBMCs	No prior antigen stimulation required	Restricted to available peptide/HLA combinations	Primarily CD8+	Hours to ~1 day	GMP-compatible	Very rapid; highly specific; reversible Streptamer technology preserves cell function	HLA restriction and reagent availability; low precursor frequencies may limit yield	Clinical application particularly for CMV; extended to EBV and AdV
CD154-based selection	Donor PBMCs	Antigen stimulation inducing transient CD154 expression	Less dependent on predefined peptide/HLA pairs	Primarily CD4+, with limited CD8+ enrichment	Approximately 14 days for expanded products	Potentially GMP-compatible	Enables enrichment of antigen-reactive helper T cells; useful for multipathogen products	CD4+ bias; longer production compared with direct selection	Experimental/early clinical development for multipathogen-specific products
Multipathogen-specific T cell expansion	Donor PBMCs or T cells	Multi-antigen stimulation	Depends on antigen-presentation strategy	CD4+ and CD8+	~10–14 days in rapid-expansion platforms	GMP-compatible approaches available	Simultaneous targeting of multiple pathogens	More complex manufacturing and product characterization; potential antigen competition	Clinical studies targeting CMV, EBV, AdV, BKV, and HHV-6
Third-party virus-specific T cell products/banks	Healthy donor PBMCs	Direct selection or prior expansion	Requires a compatible HLA-restricted specificity	Depends on manufacturing platform	Variable; cryopreserved products allow rapid availability	GMP-compliant banking possible	“Off-the-shelf” availability; useful when original donor is unavailable or pathogen-naïve	HLA matching constraints; limited persistence; donor selection and regulatory complexity	Clinical use for refractory CMV, EBV, and AdV infections, particularly in selected centers

Abbreviations: AdV, adenovirus; APC, antigen-presenting cell; BKV, BK virus; CCS, cytokine capture system; CMV, cytomegalovirus; EBV, Epstein–Barr virus; GMP, good manufacturing practice; HLA, human leukocyte antigen; HHV-6, human herpesvirus 6; MHC, major histocompatibility complex; PBMC, peripheral blood mononuclear cell.

**Table 2:** Risk-mitigation framework for alloreactivity and GvHD in adoptive virus-specific T cell therapy.

Risk Domain	Potential Concern	Risk-Mitigation Strategy
Donor selection	Pathogen-naïve donor; high degree of HLA mismatch; low precursor frequency	Prefer virus-seropositive donors where possible; consider HLA compatibility and known restriction elements
Product specificity	Residual nonspecific/alloreactive T cells	Antigen-specific enrichment; assessment of antigen responsiveness and product purity
CD4/CD8 composition	Potentially increased alloreactivity of some CD4+ populations	Characterize CD4/CD8 composition and adapt to the clinical indication
Manufacturing process	Long-term culture may alter phenotype and potentially enrich cross-reactive clones	Minimize unnecessary ex vivo expansion when rapid selection is feasible
HLA restriction	Inappropriate recognition in third-party settings	Match relevant HLA restriction elements between product and recipient
Cell dose	Higher doses may off-target alloreactivity	Conservative starting doses and, where appropriate, dose escalation
Preclinical testing	In vitro assays may not predict tissue-specific GvHD	Perform functional alloreactivity testing where feasible
Post-infusion monitoring	Delayed GvHD or unexpected tissue toxicity	Monitor for GvHD, viral load, T cell persistence, and immune reconstitution

**Table 3:** Representative clinical studies of adoptive virus-specific T cell therapy after allogeneic HSCT.

Study	Patients	Pathogen/Indication	Donor Source	Product	Key Clinical Outcome	Persistence/Immune Reconstitution	GvHD/Safety	Main Limitations
Riddell et al. [23]	3	CMV prophylaxis after allo-HSCT	Original HSCT donor	In vitro expanded CMV-specific CD8+ T cell clones	Restoration of CMV-specific immunity; no CMV viremia or pneumonia after transfer	CMV-specific CTL responses were restored and persisted	No significant toxicity reported	Very small study; prolonged clonal expansion
Walter et al. [50]	14	CMV prophylaxis after allo-HSCT	Original donor	CMV-specific T cell clones	CMV-specific CTL responses increased; no CMV viremia or disease	Persistence of transferred clones demonstrated in 3 patients; responses declined in some patients without CD4+ T cell help	No major toxicity reported	Small early study; prolonged manufacturing; limited CD4+ T cell support
Einsele et al. [24]	8	Refractory CMV infection	Original HSCT donor	In vitro expanded polyclonal CMV-specific CD4+ and CD8+ T cells	CMV infection cleared in 5/7 evaluable patients	Supported reconstitution of CMV-specific immunity	No major GvHD signal reported	Small cohort; complex manufacturing
Feuchtinger et al. [75]	18	Chemorefractory CMV infection/disease	HSCT donor	pp65-specific IFN-γ-secreting CD4+ and CD8+ T cells	CMV cleared or markedly reduced in 15/18 patients (83%)	In vivo expansion in 12/16 evaluable patients; antiviral T cell responses persisted in some patients for >6 months	No GvHD induction or acute infusion-related toxicity reported	Small, heterogeneous cohort; limited long-term follow-up
Moosmann et al. [76]	6	EBV-associated PTLD	Donor-derived	IFN-γ capture-selected EBV-specific T cells	Complete remission in 3/6 patients	Expansion and persistence for months; 2 responses sustained >2 years	No GvHD reported; one severe non-GvHD adverse event reported	Very small cohort; concomitant rituximab
Schmitt et al. [63]	2	Recurrent CMV antigenemia/viremia	HSCT donor	CMV pp65-specific Streptamer-selected CD8+ T cells	Clearance of CMV reactivation in both patients	Rapid in vivo expansion and effector differentiation	No GvHD observed	Very small cohort; HLA/epitope restricted
Leen et al. [64]	50	Severe CMV, EBV, and/or AdV infections	Third-party donors	Banked virus-specific T cells	CR/PR in 74% overall at 6 weeks; CMV 74%, AdV 78%, and EBV 67%	In vivo expansion observed; persistence varied between patients	No immediate infusion toxicity; de novo GvHD in 2/50 patients	HLA matching constraints; limited pathogen coverage; heterogeneous products
Gerdemann et al. [79]	11	AdV, EBV, and CMV	HSCT donor	Rapidly generated multivirus-directed T cells	Clinical and virological responses reported across treated infections	Virus-specific T cell expansion associated with viral control	Generally favorable safety profile; limited GvHD reported	Small early study; heterogeneous patient population

Abbreviations: AdV, adenovirus; allo-HSCT, allogeneic hematopoietic stem cell transplantation; CMV, cytomegalovirus; CR, complete remission; CTL, cytotoxic T lymphocyte; EBV, Epstein–Barr virus; GvHD, graft-versus-host disease; HLA, human leukocyte antigen; IFN-γ, interferon-gamma; pp65, phosphoprotein 65; PR, partial remission; PTLD, post-transplant lymphoproliferative disease.

## Data Availability

No new data were created or analyzed in this study. Data sharing is not applicable to this article.
